# Biodiversity Offsets: A Cost-Effective Interim Solution to Seabird Bycatch in Fisheries?

**DOI:** 10.1371/journal.pone.0025762

**Published:** 2011-10-19

**Authors:** Sean Pascoe, Chris Wilcox, C. Josh Donlan

**Affiliations:** 1 CSIRO Marine and Atmospheric Research, Brisbane, Queensland, Australia; 2 CSIRO Marine and Atmospheric Research, Hobart, Tasmania, Australia; 3 Advanced Conservation Strategies, Midway, Utah, United States of America; 4 Department of Ecology and Evolutionary Biology, Cornell University, Ithaca, New York, United States of America; Swansea University, United Kingdom

## Abstract

The concept of biodiversity offsets is well established as an approach to environmental management. The concept has been suggested for environmental management in fisheries, particularly in relation to the substantial numbers of non-target species—seabirds in particular—caught and killed as incidental bycatch during fishing activities. Substantial areas of fisheries are being closed to protect these species at great cost to the fishing industry. However, other actions may be taken to offset the impact of fishing on these populations at lower cost to the fishing industry. This idea, however, has attracted severe criticism largely as it does not address the underlying externality problems created by the fishing sector, namely seabird fishing mortality. In this paper, we re-examine the potential role of compensatory mitigation as a fisheries management tool, although from the perspective of being an interim management measure while more long-lasting solutions to the problem are found. We re-model an example previously examined by both proponents and opponents of the approach, namely the cost effectiveness of rodent control relative to fishery area closures for the conservation of a seabird population adversely affected by an Australian tuna fishery. We find that, in the example being examined, invasive rodent eradication is at least 10 times more cost effective than area closures. We conclude that, while this does not solve the actual bycatch problem, it may provide breathing space for both the seabird species and the industry to find longer term means of reducing bycatch.

## Introduction

Biodiversity offsets are increasingly being applied in the terrestrial environment in order to balance development with environmental conservation and to compensate for the residual unavoidable impacts of development projects [1], [2]. Much of the focus of biodiversity offsets has been on replacing habitats rather than individual species *per se*. In marine based industries, particularly fishing, damage is often inflicted on the species directly as well as on their habitats. Eight percent, or 7.2 million tonnes, of the global fisheries catch consists of non-target species which are subsequently discarded [3]. This mortality is having major impacts on species and ecosystems [3], [4], [5], [6], [7].

Fisheries management generally attempts to minimize these impacts through either technical measures (e.g. turtle excluder devices on trawl fisheries to minimize turtle catch) or, where suitable technical measures are unavailable, through preventing access to areas where a high probability of contact with species of conservation interest exists. In some cases, fishing gear modifications and other low-cost measures are effective in reducing bycatch for some species and are being implemented [8], [9], [10]. However, in other cases avoiding unacceptable levels of mortality has proven difficult, and costly regulatory interventions are becoming commonplace. For example, New Zealand's squid and Hawaii's swordfish fisheries have both been recently closed due to bycatch of endangered marine vertebrate species [11], [12], [13].

The imposition of technical measures and closures impose costs on the industry, with the latter in particular potentially being substantial. In a limited number of cases, the potential for offsets exist that may enable species protection to be maintained without imposing substantial costs on the industry through closure, and provide a “breathing space” for both the fishing industry and the species until some longer term mitigation measure can be developed.

Of primary concern in this paper is the case of seabirds, the incidental catch of which is taken by pelagic fishing fleets such as those that target tuna and squid. However, seabird species that are impacted by fisheries bycatch spend part of their life on land. Events in these terrestrial habitats often lead to significant mortality. For instance, while fisheries bycatch is affecting seabirds globally, particularly albatross, petrels, and shearwaters [14], of greater concern in many instances is the impacts of invasive mammals on breeding colonies. Invasive predators such as feral cats (*felis catus*) and rats (*Rattus* spp.) have decimated seabird breeding colonies worldwide, preying on eggs, chicks, and adults of many species [15], [16]. Invasive rodents have been introduced to over 82% of the world's major island groups ranging from the Arctic to the Antarctic, and feral cats occur on most of the world's islands, including Australasia and the islands of the Atlantic, Pacific, and Indian Ocean [15], [17]. Three-quarters of seabirds listed by the IUCN are threatened by invasive species, compared to 47% threatened by fisheries bycatch [18]. Indeed, invasive mammals are responsible for most vertebrate extinctions over the past six centuries, the overwhelming majority occurring on islands [19], [20].

Despite the threat posed by invasive mammal predators to many seabird species, research and management is often directed at anthropogenic sources of mortality such as bycatch. Measures such as closures, restrictions on fishing activities, and gear modifications are aimed at addressing the externality directly, generally resulting in higher costs to the industry and in many instances, lower revenues. While bycatch reduction technology is improving, experimental results are often not translated into actual bycatch reductions in the fishery unless substantial compliance and enforcement measure are introduced [9], all at additional cost to the industry.

For at least some seabird species, greater reductions in mortality (and hence greater increases in benefits) could potentially be achieved by diverting resources from the fishery to other conservation activities. Such alternative measures may include the eradication of invasive species [21], [22] or the creation of new (or restoration of old) breeding habitats. This is akin to the concept of biodiversity offsets used in environmental management in other industries.

The potential for biodiversity offsets as a fisheries management option has received mixed, and mostly adverse, responses. Initial proposals [21], [22] received severe criticism, with claims that it may do more harm than good if it diverts attention from the bycatch issue directly [23], that the model used in the analysis was flawed [24], [25], or that it is limited in its application to only part of the bycatch problem [18]. These criticisms were largely focused on the assumption that biodiversity offsets may replace the need for bycatch reduction. However, when a species is under threat and bycatch reduction technologies are not sufficient to address the problem, biodiversity offsets may be sufficient to “buy time” for the species while longer term solutions are sought [26]. If the only other feasible remedial measure is a fishery closure, then biodiversity offsets may be a viable option for fisheries management, even if only as a stop-gap measure while bycatch issues are addressed more fully.

In this paper, we examine the potential ecological and economic benefits that may arise through the adoption of a biodiversity offsets approach to the management of bycatch of non-market, but nevertheless valuable, species in fisheries. An example is presented of a potential application of such an approach to seabird conservation. We build on previous modeling work of a colony of seabirds that has interactions with both fishers and an invasive species [22], taking into account the key criticisms raised with this earlier work [24], [25]. We compare the relative costs and conservation benefits of a fishery area closure and invasive species eradication (an offset system), allowing for the possibility of technical solutions to the bycatch problem also to be developed over time. We also examine the incentive structures that each system creates and the effect of this on long term conservation and economic impact on the fishing industry. We find that an offset system may be more cost effective than a closure as an interim measure while longer term solutions are being developed.

The next section provides background to the case study. This is followed by an overview of the model used in the analysis and results of the different scenarios examined. Finally, the implications of the results for the potential use of biodiversity offsets for seabird conservation are discussed.

### Seabird bycatch in the eastern tuna and billfish fishery

The Eastern Tuna and Billfish Fishery (ETBF) operates along the entire east coast of Australia, extending to (and in a few small areas, beyond) the Australian exclusive economic zone. The fishery targets four tuna species (yellowfin, bigeye, skipjack and albacore) as well as several billfish species. In 2008–09, the total value of landings from the fishery was estimated to be around AU$39m [27], taken by 57 active vessels [28]. The fishery is currently managed through individual transferable catch quotas on the key target tuna and billfish species, implemented in March 2011. Prior to this, the fishery was managed through an individual transferable effort unit system based on the total number of hooks that could be deployed. In 2005 and 2006, a Commonwealth Government funded buyback program aimed at removing excess capacity in Australian fisheries reduced the fleet from 113 to the current 57 active vessels [28].

**Figure 1:**
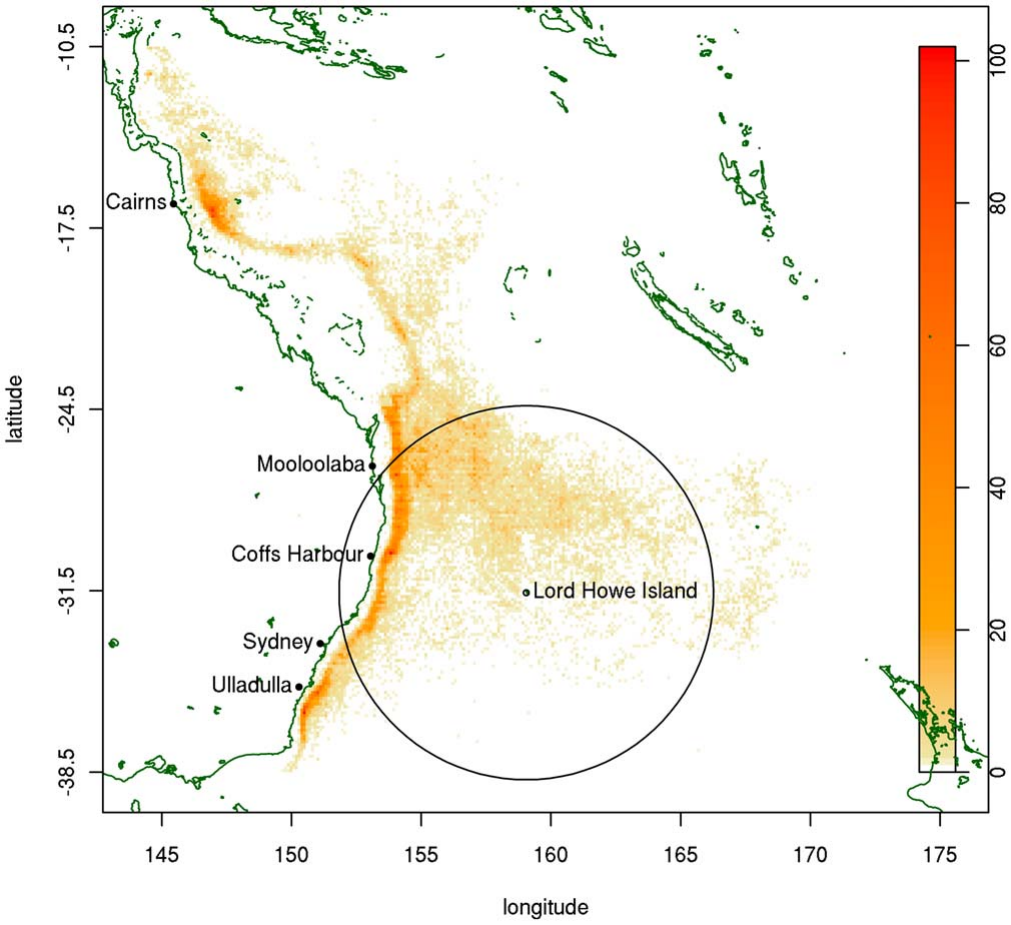
Distribution of total fishing days in the ETBF, 2003–08, and observed foraging range of shearwaters. The color represents the intensity of fishing in terms of number of days fished. Lord Howe Island lies at the centre of the range.

As with many longline fisheries, the incidental bycatch of seabirds is a problem. Flesh-footed shearwaters (*Puffinus carneipes*) suffer the greatest mortality, estimated at 1800–4500 birds per annum [29], although there is considerable discrepancy between “official” estimates of seabird bycatch from the fishery [30] and estimates derived from other studies (e.g. [29], [31], [32]). The east coast population breed exclusively on Lord Howe Island (off the New South Wales north coast) [33], with foraging seabirds covering distances of up to 800 km from the Island [34]. Studies of foraging behavior found that over half the foraging sites overlapped with tuna vessels, with most of this overlap occurring in areas of highest fishing activity between the Island and the mainland coast [34] (Figure 1). Although the total fleet size has been substantially reduced since 2005, much of the reduction has taken place in the northern and southern extremities of the fishery, so the impact of the restructuring on shearwater bycatch has been less substantial. The incidental catch (or bycatch) of seabirds during longline fishing is still listed on ‘Schedule 3 Key Threatening Processes’ of the *Endangered Species Protection Act 1992* (www.environment.gov.au/biodiversity/threatened/ktp/longlinefishing.html).

A management objective has been introduced to reduce seabird bycatch to a target rate of less than 0.05 birds per 1000 hooks deployed [35]. In comparison, existing bycatch rates were estimated to average 0.15 birds per 1000 hooks for the fishery as a whole, and average 0.779 shearwaters per 1000 hooks in waters surrounding Lord Howe Island (between 25°S and 35°S) [31]. A number of measures have being trialed to reduce seabird bycatch as part of a bycatch action plan. These include prohibition of setting longlines during daylight hours, the use of heavily weighted lines, and the use of underwater setting chutes. While the measures have reduced bycatch of seabirds in the fishery [30], mortality rates still far exceed the target rate and bird populations are still expected to decline [31]. Further, they are costly to the industry to implement, may pose health risks to operators (several severe injuries and one death have been recorded as a result of using the new gear), and are difficult to enforce.

With the entire eastern Australian population of flesh-footed shearwater breeding on Lord Howe Island and evidence of a population decline [29], fishery area closures may be implemented, with temporary closures already having been implemented in recent years. Based on fishery observer data on bycatch rates with distance from the nesting colony on Lord Howe, a closure adequate to achieve the Environment Australia (1998) bycatch target would require an area closure of 785 km radius around Lord Howe Island, consistent with the observed foraging range [34]. This includes much of the area of high activity in the fishery, and may result in significant losses in total revenue. Such extensive closures have been implemented elsewhere for bycatch reduction purposes. For example, an even larger area was closed to longline fishing around Hawaii between 1999 and 2004 in order to reduce bycatch of turtles [36].

Bycatch is not the only threatening process for the seabirds, and demographic impacts on flesh-footed shearwaters occur from on-island threats such as habitat loss, ingestion of plastic, and predation by invasive predators [29]. Rat control is currently undertaken on the Island through poisoning, but evidence of damaged eggs consistent with rat predation persists. While the actual impact of rat predation is debatable [24], previous modeling work suggests that even modest predation rates may be having a significant impact on the seabird population [37].

## Methods

To illustrate the potential role of biodiversity offsets in fisheries, we conducted a simple bio-economic analysis of a measure to offset bycatch of flesh-footed shearwaters in the ETBF. Several previous biological models of the seabird population on the island have been developed [22], [25], [29], [31], [37], although there has been considerable criticism [24], [25] of the original model used to illustrate the potential benefits of biodiversity offsets for the island [21], [22]. The model used in this analysis is based on an age-structured population model developed by Baker and Wise [31], and is equivalent in characteristics to the model suggested by Finkelstein et al [37]. Further, the key parameter estimate changes proposed by Finkelstein et al [37] are also adopted, although alternative scenarios of rat predation are also considered. The model was run over a 40 year period.

### Population model description

The model used was a simple population dynamics model, adapted from Baker and Wise [31]. The number of adult birds in age class 7≤i≤40 in year t>1, *N_i,t_*, is given by
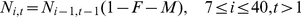
(1)where *F* is the rate of fishing mortality due to bycatch and *M* is the rate of natural mortality. The maximum age of the birds was assumed to be 40 years. The original model [31] was based on the average number of female fledglings per female, and the population was modeled in terms of female numbers. As the sex ratio is approximately 1∶1 [31], the model is effectively based on pairs of breeding birds rather than individual birds.

The number of fledglings in time *t*, *N_1,t_* is given by
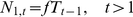
(2)where *f* is the average number of fledglings per pair (the effective fecundity rate), and *T_t_* is the total number of adult breeding pairs (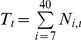). The effective fecundity rate takes into account natural mortality of eggs and chicks, including predation mortality, and is defined by
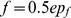
(3)where *e* is a constant that represents the average number of eggs per breeding pair and other factors affecting egg production and *p_f_* is the probability that the egg will hatch and the chick survive to become a fledgling. Only half this product is taken as two chicks are required to survive to form one new breeding pair. The original model specified fecundity directly as *f* = 0.263 [31] based on a study of the Lord Howe Island population, whereas the analysis of Finkelstein et al [37] and Wilcox and Donlan [22] assumed that fecundity was a function of a range of parameters, one of which was the probability that the chick would hatch and survive to become a fledgling (i.e. *p_f_*). The constant in equation (3) was estimated given the fecundity estimate from Baker and Wise [31] and the probability of survival from Finkelstein et al [37].

The number of juveniles, *N_i,t_*, where 1<*i*<7, was subject to density dependent survival, 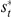, such that
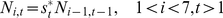
(4)Following Baker and Wise [31], the density dependent survival rate was given by
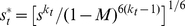
(5)where *s* is the base rate of juvenile survival, and *k_t_* is a density dependent parameter relating to changes in the population size relative to the first year, given by
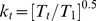
(6)


The initial number of breeding pairs in each age class in time 1 was estimated based on the total number of birds, and the observed mortality and survival rates within the different ages (Table 1). The simulations were undertaken by varying the values of *p_f_* and *F*. The effect of removing rats from the island is uncertain, although there is strong evidence to suggest that predator removal can increase productivity of ground nesting seabirds [38]. The original analysis by Wilcox and Donlan [22] assumed that breeding success (the ratio of eggs to fledges, initially estimated at 0.513 in the baseline analysis) would increase to 1 as a result of removal of rat predation [22]. Rat consumption rates were estimated using allometric relationships for metabolic estimates and reported rat weights from islands [39], [40], and the average of historic reports of unmanaged rat densities of 45.5 per ha (range 4–94 rats/ha). They further assumed rats met 100% of their daily metabolic requirements from seabird eggs and chicks, when available. Their resultant estimated change in chick survival is consistent with other empirically based studies. For example, reducing the rat population by around 57% resulted in a 61% increase in the breeding success of shearwaters in the Mediterranean [41]. Extrapolating from this, complete removal of rats could be expected to result in a 100% increase in breeding success. However, as the rat population is not entirely unmanaged, the assumed current rat density is likely to have been overestimated, as the original study was intended more to illustrate the potential hypothetical benefits of biodiversity offsets than provide a definitive cost-benefit analysis of the eradication program on the Island. In this analysis, we use an upper (0.831) and lower (0.748) estimate of breading success observed in shearwater populations on predator free islands [37].

**Table 1:** Model parameters used in the simulations.

Variable		Baseline	Closure/bycatch reduction	Rat eradication	Rat eradication and bycatch reduction
Total number of breeding pairs in period 1	*T_1_*	17462	17462	17462	17462
Base rate of juvenile survival	*s*	0.766	0.766	0.766	0.766
Natural mortality rate	*M*	0.06	0.06	0.06	0.06
Constant relating to egg production (derived)	*e*	1.027	1.027	1.027	1.027
Probability that egg survives to fledgling	*p_f_*	0.513	0.513	0.748,0.831	0.748,0.831
Fishing mortality rate	*F*	0.079	0.006	0.079	0.006

### Estimating costs and benefits

The potential economic impact of a closure of the size required to achieve the target bycatch rates was estimated using a spatial simulation model of the fishery developed for a separate study aimed at comparing an incentive based management approach to area closures [42]. The model is based around a multinomial logit model of fisher location choice, and includes information on the cost structure of the fleet [28]. The location choice model was estimated based on trip level data to areas in the fishery defined by a one degree grid. The model included the value per unit effort in each fishing area, a cost proxy consisting of the fuel price times the distance to the area from the vessels' home ports, and previous levels of fishing activity in the area (both of the individual vessel and the fleet as a whole). Closing an area was simulated by removing it from the choice set. For this study, all fishing areas within the foraging range of Lord Howe Island were removed from the choice set.

Given that the fishery is based on a mobile resource that has a different spatial stock structure from year to year, the model was run using two different years of trip level data (i.e. catch rates relating to 2004 and 2007) but applied to the fleet operating in 2007. The model was used to estimate where fishers may relocate their fishing effort given the closure, from which changes in revenue (based on differential catch rates), change in crew costs (based on changes in revenue) and changes in fuel costs (based on changes in distance travelled) were estimated. The analysis assumed that all vessels would undertake the same number of trips as observed in 2004 and 2007, although their fishing location choice would be affected by the closure. Hence, fixed costs would remain the same, and change in profits would be a function only of change in revenue and variable costs. As such, it may overestimate the costs as in some instances fishers may choose not to fish given the closure.

A cost benefit analysis requires both the outcomes and the costs of the alternative mitigation measures to be valued in monetary terms. However, as the value of the ecological outcomes is unknown, and it is beyond the scope of this example to derive such values, cost effectiveness analysis is used to determine the most efficient mitigation measure. Cost effective analysis is increasingly being used to assess the relative benefits of alternative conservation policies when valuing benefits is difficult or unacceptable [43], [44], [45]. An implicit assumption, however, is that the value of the stock recovery is considered by society to exceed the costs. Hence, the least cost method to deliver seabird population recovery is considered the most efficient. Given that the objective is also to eliminate the problem in the longer term, we aim to explore cost-effective ways of keeping the fishery operational while securing the existence of the seabird population until a means of eliminating the bycatch problem can be developed.

Determining an appropriate ecological outcome for the purposes of comparing the costs is not straightforward. Cost effectiveness analysis utilizes an output measure that is not measured in monetary terms, but is believed to be proportional to the utility derived from its production. For simplicity, we assume more seabirds are preferred to less, and that the marginal value of a seabird is constant, such that the increase in the number of birds reflects the value of the mitigation activity. In reality, the marginal value of an additional animal is likely to decrease with increasing population size [46], [47]. However, information on how these values may change is not available. This is a common problem with conservation values for wildlife [48], so is not unique to the case in hand. Also for simplicity, we take the incremental change in the seabird population in year 40 relative to that estimated in the baseline simulation (i.e. no management change) as our output measure. This ignores the potential time preference relating to seabirds, in that a closure “produces” more additional seabirds earlier than the rat eradication program.

Given that the costs (monetary costs to the industry) and benefits (increased seabird numbers) occur with different magnitudes at different points over time, these future costs and benefits are converted into a net present value for comparison between management options. The choice of an appropriate discount rate in such a case is complex, and there are many arguments for the use of a low discount rate when measuring changes in values of environmental assets over time, particularly when the resource is non-renewable or the environmental impacts effectively irreversible in a reasonable time frame [49]. Some economists argue that the discount rate should decline over time to attach greater weight to the welfare of future generations, particularly when negative externalities may necessitate increased environmental expenditures over time [50] or uncertainty about future outcomes is high [51]. Others argue that resource scarcity in the future will increase the value of the environmental asset and a more appropriate approach is to factor in these higher values and discount using an unmodified social discount rate [52], [53].

The net present value in this study was estimated using both a 5% and 10% discount rate, consistent with the range of discount rates applied in Australian fisheries management [54] and implicit in fisher decision making [55], [56]. While these discount rates appear relatively high given the conservation orientation of the study, they are applied only to the costs imposed on the commercial industry rather than society as a whole. We do not “discount” the number of seabirds that are generated in the future as a result of the options, so consider a seabird in the future to have the same value as a seabird now. Such an approach has been used elsewhere when looking at the cost effectiveness of options for preserving endangered species. For example, in the case of the northern spotted owl, the costs to the industry were discounted at their “normal” rate and the output measure was the probability of survival, linked to future population estimates but not discounted [57].

### Scenarios

For the purposes of comparison, we estimate the costs to the fishing industry and impacts on the seabird population of eliminating bycatch mortality through a large scale area closure and increasing chick survival rates through eliminating rats on the island. The effects of the closure on the seabird population were estimated by assuming catch rates declined to the target of 0.05 birds per thousand hooks. Catch rates are a function of both the catchability and availability of the stock, the latter being substantially lower outside the area of the closure. The effect of this on the population was simulated by reducing the rate of fishing mortality of seabirds from its previous level of 0.096 [31] to 0.006 [25], assuming the target catch rate is achieved.

In the previous analyses [21], [22], both the closure and rat eradication scenarios implicitly assume that there are no improvements in gear technology over the 40 year period. This assumption is unrealistic, and it is likely that the “bycatch problem” can be solved (or at least substantially reduced) during this period. The analysis allowed for the possibility of technology to reduce bycatch to the required level after 5 years and after 10 years. In terms of the closure scenario, this effectively meant that the costs of the closure were only incurred for the period that the closure was necessary, with no other impact on the seabird population (as the fishing mortality had already been reduced due to the closure). For the rat eradication scenario, fishing mortality was reduced to the same level as the closure after 5 and 10 years respectively. The costs of any new technologies in terms of equipment cost and change in catch rates were not considered as these would be equally applicable to both the closure and rat eradication scenarios.

## Results

### Changes in seabird population

**Figure 2:**
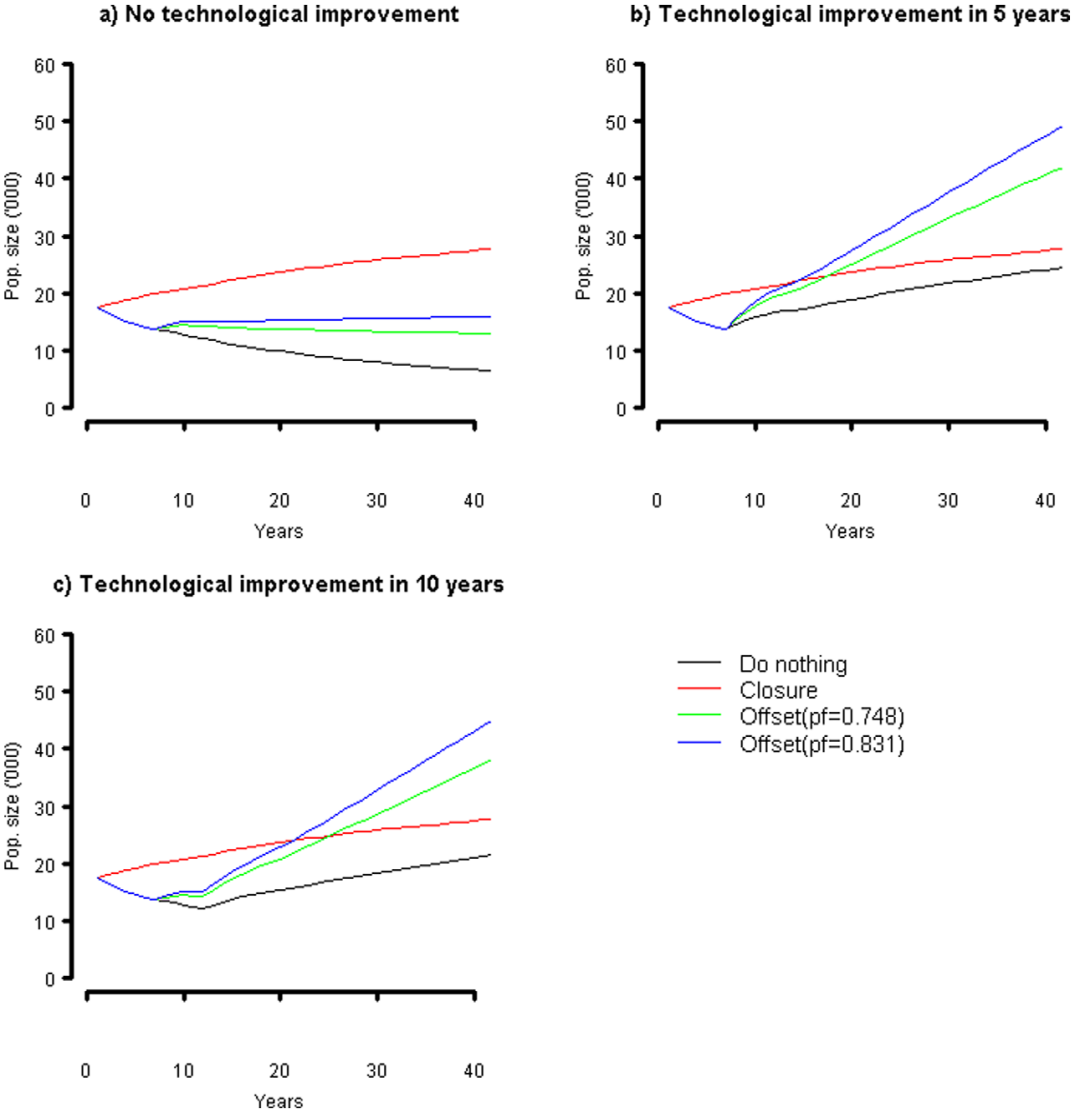
Change in seabird population over time a) no gear improvements; b) gear improvements after 5 years; c) gear improvements after 10 years.

The impact of the different options and survival rate scenarios on the seabird population is illustrated in Figure 2, and summarized in Table 2. For comparison, the projected change in population if no mitigation measures are implemented is also presented. Rat mortality is assumed to continue in the closure scenario, while current fishing mortality is assumed to continue for the rat elimination option. The assumption about technology changes is applied to all scenarios equally (including the do-nothing scenario that will also benefit from the improved bycatch reduction technology). For the closure scenario, it is assumed that the fishery will reopen if technical solutions are found, but that there is no subsequent change in seabird mortality (as the closure is assumed to achieve the same low mortality rate as the improved fishing gear).

**Table 2:** Estimated numbers of seabirds under different scenarios after 40 years.

	Baseline	Closure	Rat eradication	
	(Do nothing)		*p_f_* = 0.748	*p_f_* = 0.831
Population after 40 years				
• no gear improvement	6385	27831	12934	15900
• improvements in 5 years	24432	27831	42026	49217
• improvements in 10 years	21601	27831	38130	44986
Increment against baseline				
• no gear improvement		21445	6549	9515
• improvements in 5 years		3399	17594	24785
• improvements in 10 years		6229	16529	23384

Note: *p_f_* is the probability that an egg will eventually become a fledgling.

From Figure 2, the time trajectory of recovery for the conservation actions differs. Closing a large area of the fishery results in an immediate reduction in adult mortality due to the reduction in seabird bycatch. With more birds of breeding age surviving each year, the population starts to increase immediately. In contrast, reducing chick mortality through eradicating rats has no impact on the breeding population until they reach maturity, so no immediate improvement in the population is observed (Figure 2). However, once these juveniles reach maturity, the population is expected to increase relative to the do-nothing scenario. A key observation from Figure 2 is that rat eradication, even at the most pessimistic outcome in terms of egg-to-fledgling survival, is likely to stabilize the population, albeit at a lower level than the starting point. This suggests that, at worse, the bycatch from the fishery is sustainable if the land-based source of mortality is removed.

An early introduction of bycatch reduction technologies (e.g. after 5 years) greatly reduces the benefits of a closure in the longer term (relative to the benchmark scenario that also experiences the technological improvement), but enhances the benefits of the rat eradication. In the analysis, rat-related mortality is assumed to continue under the closure scenario, and this continues to depress the population growth.

### Costs of Mortality Reductions

Based on the location choice model results, the economic impact of the closure (estimated as reduction in fishery profits) is very specific to the underlying stock distribution, ranging from $0.6m (under 2004 stock conditions) to $2.2m (under 2007 stock conditions) in 2009–10 dollars. To place this in context, the total economic profits in the fishery in 2007–08 was estimated to be only $2m [28].

In contrast, the cost of eradicating ship rats and mice from the Island has been estimated to be only AU$0.92m (in 2009–10 dollars) [58]. These costs, while appearing relatively low, are consistent with rodent eradication costs experienced elsewhere [59]. This is a one-off cost, and assumes that re-infestation does not occur. The monitoring and surveillance costs of ensuring that re-infestation does not occur are unknown, and are likely to be borne by vessels visiting the island. As a result this is most likely an underestimate of the true cost of rat eradication. Similarly, some form of incentive will need to be introduced on the fishers to ensure that technical solutions are sought to reduce bycatch. A range of options exist, including, for example, a bycatch tax or quota [60]. The cost of these incentive based systems on the industry is not explicitly considered in the analysis, but the implications of these are discussed in later sections of the paper.

The net present value of the costs of each option under the different scenarios is given in Table 3. As only the closure has ongoing costs, discounting this flow of costs at a high discount rate results in a lower present value of the cost stream from this option than if a low discount rate was assumed. In other words, with a low discount rate the total cost of the closure would be substantially higher. Hence, we are artificially favoring the closure to some extent in the analysis by reducing the costs imposed. In contrast, the costs of the eradication program occur all in the first year so are not discounted over time.

**Table 3:** Net present value of the costs of different options.

			Discount rate					
			5%			10%		
			Closure length			Closure length		
	Up-front cost	Annual Cost	40 year	5 year	10 year	40 year	5 year	10 year
Closure - lower estimate	-	0.64	$11.0	$2.8	$4.9	$6.3	$2.4	$3.9
Closure - higher estimate	-	2.34	$40.2	$10.1	$18.1	$22.9	$8.9	$14.4
Rat eradication	$0.92	-						

The cost of the closure option was varied depending on the length of time required for a technological solution to the bycatch problem to be introduced. For example, in the scenario where the technical solution is achieved after five years, the closure and its associated costs are assumed to remain in place only for the first five years. As the time required to achieve a technical solution is unknown, two time periods were examined – five years and ten years.

### Cost effectiveness of the measures

The cost effectiveness ratio was estimated as the average cost per additional seabird relative to the baseline scenario. From Table 4, the rat extermination is the most cost effective method of maintaining or recovering the seabird population. The cost per bird further decreases if bycatch can also be removed though new technologies, whereas new technologies effectively increase the cost per bird under a closure scenario. While this result appears counter intuitive, the benefits of the closure if gear modifications are introduced relatively early are limited (Figure 2), whereas the gear modifications complement the reduced predation mortality in the rat eradication option.

**Table 4:** Cost effectiveness of the alternative options ($/seabird), derived by dividing the number of seabirds after 40 years by the cost of the management measure.

	Closure		Rat eradication	
	Low cost	High cost	*p_f_* = 0.748	*p_f_* = 0.831
5% discount rate				
• no gear improvement	519	1897	142	98
• improvements in 5 years	812	2970	54	38
• improvements in 10 years	784	2866	57	41
10% discount rate				
• no gear improvement	296	1081	142	98
• improvements in 5 years	711	2600	54	38
• improvements in 10 years	624	2281	57	41

## Discussion

The example above illustrates that, if conservation of species impacted by fishing is an objective, then non-fishing related options may be more cost effective than limiting fishing activity, particularly if technical solutions are likely to be found to reduce bycatch in the near future. In this case, conservation benefits could be achieved at lower cost if the fishing industry funded the rat eradication through a bycatch levy, for example, rather than ceased fishing in the area. Such a levy would also have the additional benefit in that it would create an incentive for fishers to avoid bycatch of seabirds [60], and could also provide funds for research into new fishing gear to further mitigate the problem. This is not to say that the externality imposed by fishing should not be eliminated or reduced, and indeed the analysis assumes that it will be reduced at some point. Bycatch is a highly visible form of mortality, and pressures on the industry to reduce bycatch will persist even if offset actions are undertaken. However, offset activities may give the fishing industry “breathing space” in which new technologies can be developed that are themselves cost effective in reducing the bycatch problem. It also provides an opportunity for the fishing industry to engage with conservation groups, and be seen to be concerned about the ecological problems that it is contributing to.

The example assumes a one-off program to eradicate rats from the Island. No doubt, on-going monitoring of both rats and seabirds will be required to ensure that the eradication has been successful and that seabird stocks are recovering. This will be particularly important given that recovery may not be obvious for several years. Given that the alternative – closure of the fishery – imposes far greater costs on the industry, then fishers have an incentive to continue paying a levy for continuing mitigation related activities, effectively as an insurance premium against closures.

Alternatively, given the potential difficulties in enforcing such a levy (as considerable incentives not to report bycatch would exist), a fee for fishing in the interaction area could also potentially be imposed. This fee could potentially be linked to previous seabird encounters, with areas of high risk attracting a high fishing fee. This would create some incentive to avoid bycatch through the initial decision to fish in the area or not, and would provide funds for the offset and research into other mitigation measures. Vessel monitoring technology is already in place to track vessel location, so monitoring and enforcing such a levy would not be excessively expensive. Given that a closure could cost the industry between $0.6m and $2m a year, then there is considerable benefit to both industry (in terms of lower costs) and society (in terms of a larger seabird population) in implementing some form of bycatch levy to fund offsetting activities even if monitoring and compliance costs are moderate.

An issue that the analysis has not addressed is the disutility associated with the bycatch itself. In short, people do not like the fact that some animals are unintentionally killed as a byproduct of their food production, even more so for species that are seen to be iconic. Although the offset option is estimated to result in a higher population of seabirds than the closure *ceteris paribus*, it is also likely to result in an overall increase in seabird mortality; at least until bycatch reduction technologies are available. This disutility is difficult to measure, and the extent to which the non-market value of a larger and more secure population offsets the cost of higher bycatch mortality is unknown. With a decreasing marginal value of seabirds as the population increases, at some point the cost of the higher bycatch may exceed the benefits of the larger population. This reinforces the need to have some form of incentive system to ensure that measures to avoid or reduce bycatch area adopted as rapidly as possible.

The results of the analysis are also consistent with those of Finkelstein et al [37], who found that combining rat eradication with bycatch reduction resulted in the greatest increase in population size. What mainly differs is the interpretation of the results. Finkelstein et al [37] concluded that as bycatch reduction has a bigger impact than rat eradication when viewed separately, bycatch reduction should be the priority option, effectively discounting the potential role of biodiversity offsets. They also conclude that cost should not be a consideration when protecting species [25]. Here, we have demonstrated that biodiversity offsets can achieve conservation objectives at a considerably lower cost than draconian measures such as closures, and can provide greater conservation benefits if used with bycatch reducing technologies than the latter alone.

We have focused this study only on rat eradication, along the lines of the original studies. However, the principles could be extended to different offsetting approaches such as habitat restoration or creation. Restoration activities are currently underway on several islands off the New South Wales coast with the aim of rebuilding seabird colonies (some also in conjunction with invasive species eradication) [61]. Elsewhere, new habitat creation has been successful in offsetting the impacts of port development on seabird populations [62].

### Conclusions

The key critics of the original studies in this area [21], [22] argued that bycatch was the major threatening process for seabirds, and that biodiversity offsets did not address this problem [18], [24], [25]. This point is not refuted in this paper, and the model simulations also support the benefits of rapid adoption of bycatch reduction technologies. However, like world peace, bycatch elimination cannot be achieved over night. If the technologies currently existed to eliminate seabird bycatch then no doubt they would be in place already, at least in some fisheries. Further, technology alone cannot solve the bycatch problem without effective enforcement and governance also being in place. Biodiversity offsets may play an important role as a “stop-gap” measure to provide initial relief for at least some seabird populations that have threats other than fishing affecting their populations also. While these may not be an appropriate long term solution, they may prevent more drastic and costly measures (e.g. fishery closure) being introduced while more suitable technologies are being developed.

When designed under the proper framework, biodiversity offsets require a mechanism for generating revenue from common pool resources that can be transferred to support high impact conservation actions. Returning to the fisheries context, using individual vessel levies for bycatch 1) provides regulatory certainty for operators, an essential ingredient for effective businesses; 2) creates individual incentives for fishers to avoid bycatch; and 3) could fund mitigation actions that at least partially offset the bycatch that does occur; and potentially fund research into the development of bycatch reduction technologies. Unlike other offset programs that have been criticized as leading to reductions in environmental quality, for example through substituting lesser (ecologically) valued wetlands to those removed, fishers have an incentive for the mitigation actions to achieve outcomes greater than, or at least equal to, that of the alternative – closure.

As opposed to command-and-control approaches (e.g., fisheries closures), incorporating market externalities into the costs of fishing allows fishers an opportunity to develop innovative ways of avoiding bycatch, and a derived demand for the development of new technologies to assist in bycatch reduction. The lack of such opportunity is a common complaint in the fisheries sector, and individual incentives have been shown to be the single most important factor determining the sustainability of fisheries [63]. Further, biodiversity offsets would have significant marginal benefits since not only bycatch species but an entire suite of species, and frequently entire ecosystems, would benefit from the removal of invasive mammals and other on-island restoration actions. Biodiversity offsets provide an opportunity to constructively address a global conservation concern, and forge an alliance between conservation and fisheries organizations.
